# Malignant Potential of Gastrointestinal Cancers Assessed by Structural Equation Modeling

**DOI:** 10.1371/journal.pone.0149327

**Published:** 2016-02-18

**Authors:** Haruo Shimizu, Yoshiaki Arimura, Kei Onodera, Hiroaki Takahashi, Satoshi Okahara, Junichi Kodaira, Hirokazu Oohashi, Hiroyuki Isshiki, Kentaro Kawakami, Kentaro Yamashita, Yasuhisa Shinomura, Masao Hosokawa

**Affiliations:** 1 Department of Gastroenterology, Rheumatology, and Clinical Immunology, Sapporo Medical University, Sapporo, Japan; 2 Department of Gastroenterology, Keiyukai Daini Hospital, Sapporo, Japan; 3 Department of Surgery, Keiyukai Hospital, Sapporo, Japan; Jawaharlal Nehru University, INDIA

## Abstract

**Background:**

Parameters reported in pathologic reviews have been failing to assess exactly the malignant potential of gastrointestinal cancers. We hypothesized that malignant potential could be defined by common latent variables (hypothesis I), but there are substantial differences in the associations between malignant potential and pathologic parameters according to the origin of gastrointestinal cancers (hypothesis II). We shed light on these issues by structural equation modeling.

**Materials and Methods:**

We conducted a cross-sectional survey of 217 esophageal, 192 gastric, and 175 colorectal cancer patients who consecutively underwent curative surgery for their pathologic stage I cancers at Keiyukai Sapporo Hospital. Latent variables identified by factor analysis and seven conventional pathologic parameters were introduced in the structural equation modeling analysis.

**Results:**

Because latent variables were disparate except for their number, 'three' in the examined gastrointestinal cancers, the first hypothesis was rejected. Because configural invariance across gastrointestinal cancers was not approved, the second hypothesis was verified. We could trace the three significant paths on the causal graph from latent variables to lymph node metastasis, which were mediated through depth, lymphatic invasion, and matrilysin expression in esophageal cancer, whereas only one significant path could be traced in both gastric and colorectal cancer. Two of the three latent variables were exogenous in esophageal cancer, whereas one factor was exogenous in the other gastrointestinal cancers. Cancer stemness promoted viability in esophageal cancer, but it was suppressed in others.

**Conclusion:**

These results reflect the malignant potential of esophageal cancer is higher than that of the other gastrointestinal cancers. Such information might contribute to refining clinical treatments for gastrointestinal cancers.

## Introduction

The popular terms ‘malignant potential’ or ‘biological malignancy’ of gastrointestinal (GI) cancers including esophageal (ECA), gastric (GCA), and colorectal cancer (CRC) are versatile to use in practice but equivocal to define in theory. Nevertheless, pathologic parameters, such as tumor depth of penetration, cancer grade, lymphovascular invasion, and lymph node metastasis, are commonly used to assess the malignant potential, such as curability by endoscopic resection in GI cancers [1–4]. However, each parameter is expected to have various clinical significances reflecting the malignant potential, such as the risk of metastatic spread. Therefore, we are unable to adequately translate and use the information from preexisting pathological reviews of GI cancer. In contrast to conventional pathological reviews [5–8], we previously suggested tumor matrilysin expression can predict the metastatic potential of pathologic stage I (pT1) colon and rectal cancers [9]. Fortunately, locoregional nodes are almost invariably the initial site of metastatic spread in pT1 GI cancers. Therefore, the development of lymph node metastasis should reflect the metastatic and partly malignant potential of the primary tumor of pT1 GI cancers. In this study, to assess the poorly defined metastatic potential, we introduce structural equation modeling (SEM) focusing on pT1 GI cancers in patients who have undergone surgery.

In general, SEM encompasses two components: measurement and structural models [10]. The measurement model specifies the relationship of the latent and observed variables, whereas the structural model identifies specific relationships among the latent variables. SEM allows testing of hypothesized direct, indirect or mediated, and total associations of latent variables to each other and between observed and unobserved latent variables. Therefore, SEM can analyze relationships among the theoretical variables of primary interest without the effects being confounded by measurement errors [11]. Furthermore, SEM allows simultaneous analysis of the effect of multiple independent variables on several dependent variables, whereas multiple regression analysis testing allows evaluation of only one dependent variable at a time. In this context, the usual terms of dependent and independent variables are less appropriate because the dependent variable in one equation might be an independent variable in another equation. For this reason, the variables in a model are called either endogenous or exogenous [11].

The premise of SEM is to determine whether a theoretical model is supported by the collected data. Thus, we propose two hypotheses in this study. Hypothesis I is that the malignant potential of pT1 GI cancers can be determined by common latent variables. Hypothesis II is that there are substantial differences in the associations between the malignant potential and pathological parameters according to the cancer origin. Our primary objective was to verify these hypotheses by SEM.

## Materials and Methods

### Study participants

We conducted a cross sectional survey involving three groups of patients with pT1 GI cancer, defined as invasion of the tumor into the submucosa, who underwent curative surgery at Keiyukai Sapporo Hospital: 217 consecutive patients with ECA (male/female = 203/14, age: 41–82 years), 192 patients with GCA (male/female = 136/56, age: 33–91 years), and 175 patients CRC (male/female = 123/52, age: 31–81). All patients were enrolled during the 10-year recruitment period from Mar 2004 to Apr 2014 (Table 1). All esophageal tumors were squamous cell carcinoma, whereas others were adenocarcinomas. Clinicopathological features were classified according to the TNM (tumor, nodes, metastasis) classification of the Union for International Cancer Control [12]. We excluded patients with multiple primary cancers, familial cancer syndromes, and idiopathic inflammatory bowel disease. All participants provided written informed consent based on the Declaration of Helsinki (1964, 1975, amended in 1983, 2003 and 2008) of the World Medical Association. The study was approved by the institutional review board of Keiyukai Sapporo Hospital and the Ethics Committee of Sapporo Medical University. Clinicopathological data were retrieved from the medical and pathological records (Table 1).

**Table 1:** Characteristics of the study population.

	ECA	GCA	CRC	*P*-value
	(*N* = 217)	(*N* = 192)	(*N* = 175)	
**Age**				**4.4E-8**
**Mean ± SD (years)**	**62.7 ± 8.4**	**67.6 ± 8.4**	**62.4 ± 9.6**	
**Range (years)**	**41–82**	**33–91**	**31–81**	
**Gender**				**3.0E-10**
**Male / Female**	**203 / 14**	**136 / 56**	**123 / 52**	
**(%)**	**(93.5 / 6.5)**	**(70.8 / 29.2)**	**(70.3 / 29.7)**	
**Size**				**1.0E-13**
**Mean ± SD (mm)**	**36.5 ± 24.7**	**37.1 ± 28.6**	**17.7 ± 8.7**	
**Range (mm)**	**8.0–200.0**	**7.0–300.0**	**4.0–58.0**	
**Depth**				**6.0E-6**
**sm1 (%)**	**30 (13.8)**	**41 (21.3)**	**47 (26.9)**	
**sm2 (%)**	**114 (52.5)**	**124 (64.6)**	**86 (49.1)**	
**sm3 (%)**	**73 (33.7)**	**27 (14.1)**	**42 (24.0)**	
**Histological grade**				**1.0E-13**
**well (%)**	**82 (37.8)**	**48 (25.0)**	**137 (78.3)**	
**mod (%)**	**129 (59.4)**	**45 (23.4)**	**37 (21.1)**	
**por (%)**	**6 (2.8)**	**99 (51.6)**	**1 (0.6)**	
**ly**				**0.006**
**n (%)**	**148 (68.2)**	**115 (59.9)**	**132 (75.4)**	
**p (%)**	**69 (31.8)**	**77 (40.1)**	**43 (24.6)**	
**v**				**9.4E-8**
**n (%)**	**204 (94.0)**	**145 (75.5)**	**132 (75.4)**	
**p (%)**	**13 (6.0)**	**47 (24.5)**	**43 (24.6)**	
**n**				**3.3E-11**
**n (%)**	**131 (60.4)**	**158 (82.3)**	**155 (88.6)**	
**p (%)**	**86 (39.6)**	**34 (17.7)**	**20 (11.4)**	
**Matrilysin expression**				**0.01**
**n (%)**	**141 (65.0)**	**103 (53.6)**	**119 (68.0)**	
**p (%)**	**76 (35.0)**	**89 (46.4)**	**56 (32.0)**	

Abbreviations: ECA, esophageal cancer; GCA, gastric cancer; CRC, colorectal cancer; n, negative; p, positive.

### Demographic data and measures

The clinicopathological features of the patients were analyzed according to pathologic parameters, such as tumor depth of penetration, cancer histological grade (well, moderately, and poorly differentiated carcinoma are abbreviated as well, mod, and por, respectively), lymphatic/vascular invasion (ly/v), lymph node metastasis (n), tumor size, age, gender, and matrilysin expression. Measurements of pathologic parameters were based on the Japanese classification of esophageal/gastric/colorectal cancer as follows [13–15]. Depth of penetration was scored as submucosal invasion 1 (sm1), sm2, or sm3, in which sm2 was defined between sm1 (slight invasion was defined when the tumor invaded the upper third of the submucosal layer) and sm3 (massive invasion was defined when the tumor invaded the lower third layer) [13]. In this study, the subclassification of sm1 to 3 was commonly adopted in both gastric and colorectal cancers as well as esophageal cancers because of analyzing simultaneously with multigroup SEMs. The differentiation grade was scored according to well as 1, mod as 2, or por as 3, ly as negative 0 or positive 1, v as negative 0 or positive 1, and n as negative 0 or positive 1. All data were retrospectively retrieved from the pathological reviews and medical records.

### Matrilysin immunohistochemistry and evaluation

Archival formalin-fixed, paraffin-embedded sections were immunostained using a standard avidin-biotin peroxidase technique using LSAB2 System-HRP (Dako, Glostrup, Denmark) as recommended by the manufacturer. The mouse monoclonal antibody was against human matrix metalloproteinase-7 (also known as matrilysin) (141-7B2; Daiichi Fine Chemical, Toyama, Japan). The simplest criterion to evaluate immunoreactivity was adopted according to an all-or-nothing principle. Matrilysin immunostaining was judged as positive provided basilar and/or cytoplasmic staining were observed at the invasive edge. Avoiding observer bias, two independent researchers (KO and KY) evaluated all immunostaining in a blinded manner.

### Data analysis

Descriptive statistics were summarized as frequency distributions for categorical data, and the means and standard deviations for continuous data. For descriptive purposes, univariate statistics were conducted using the demographic characteristics. Demographic data such as age, gender, tumor size, depth of penetration, tumor grade, ly, v, n, and matrilysin expression were correlated to examine their statistical associations (statistical significance: *P* < 0.05).

**Fig 1:**
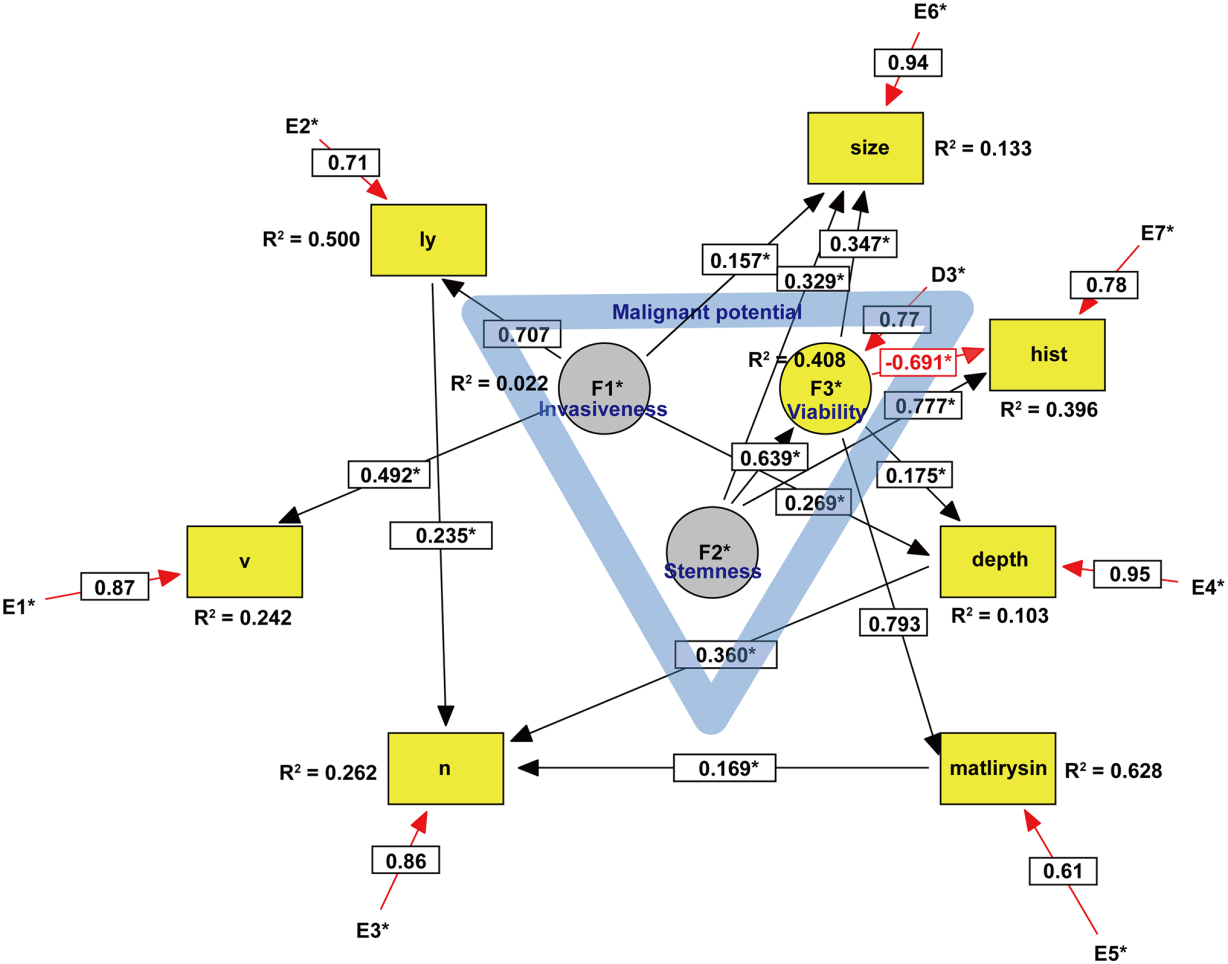
SEM of esophageal cancer. Circles indicate unobserved latent variables, while rectangles represent observed variables. Yellow indicates a dependent endogenous variable and grey indicates an independent exogenous variable. Significant paths with their estimated parameter are shown by solid lines, while insignificant paths are shown by broken lines (Fig 2). Rred arrows represent either a negative causal effect or measurement errors within the model. The blue triangle in the center suggests that the malignant potential includes three latent variables. Coefficient of determination is written as R^2^. Abbreviations: v, vascular invasion; ly, lymphatic invasion; n, lymph node metastasis; hist, histological grade.

**Fig 2:**
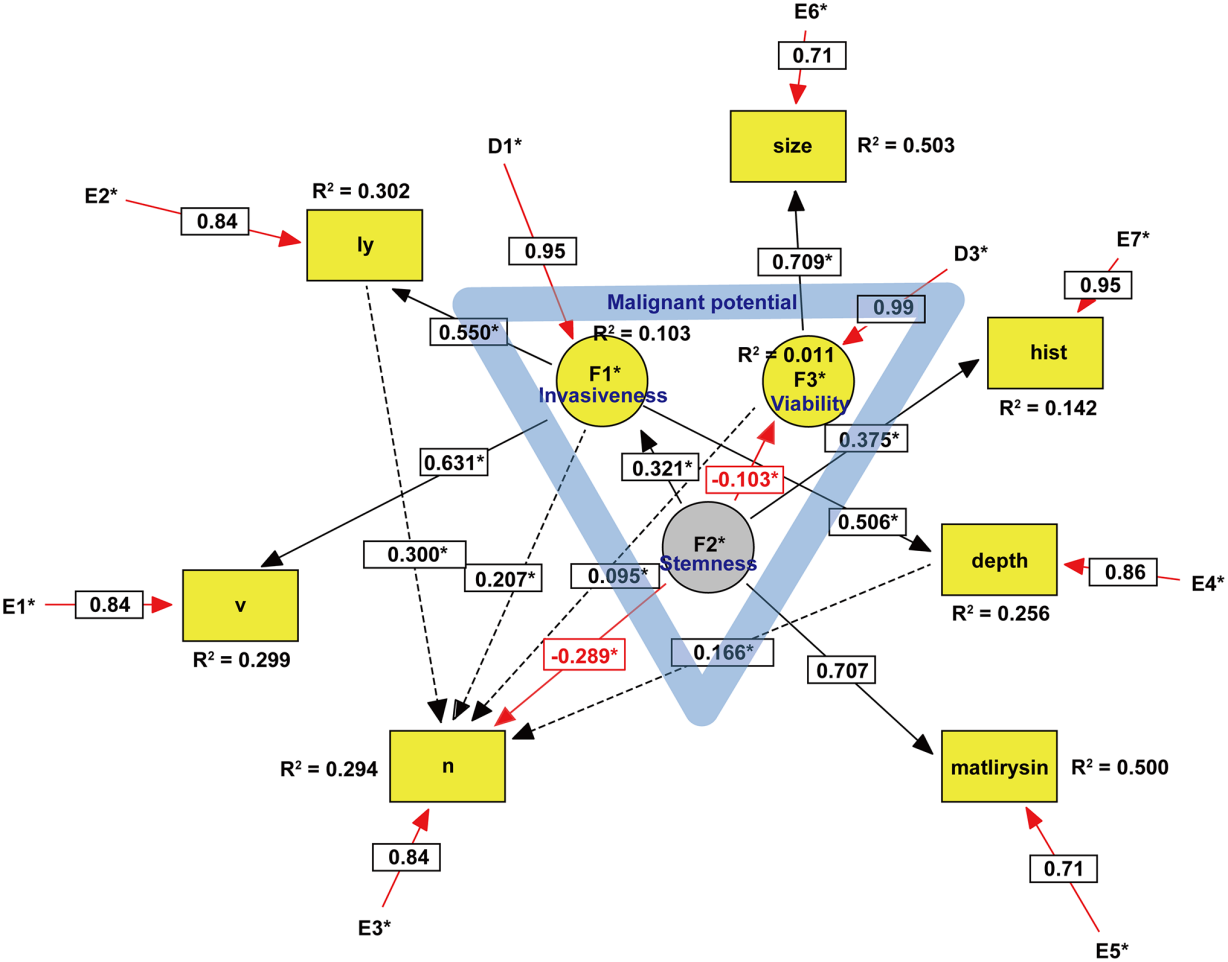
SEM of gastric cancer. Circles indicate unobserved latent variables, while rectangles represent observed variables. Yellow indicates a dependent endogenous variable and grey indicates an independent exogenous variable. Significant paths with their estimated parameter are shown by solid lines, while insignificant paths are shown by broken lines (Fig 2). Rred arrows represent either a negative causal effect or measurement errors within the model. The blue triangle in the center suggests that the malignant potential includes three latent variables. Coefficient of determination is written as R^2^. Abbreviations: v, vascular invasion; ly, lymphatic invasion; n, lymph node metastasis; hist, histological grade.

**Fig 3:**
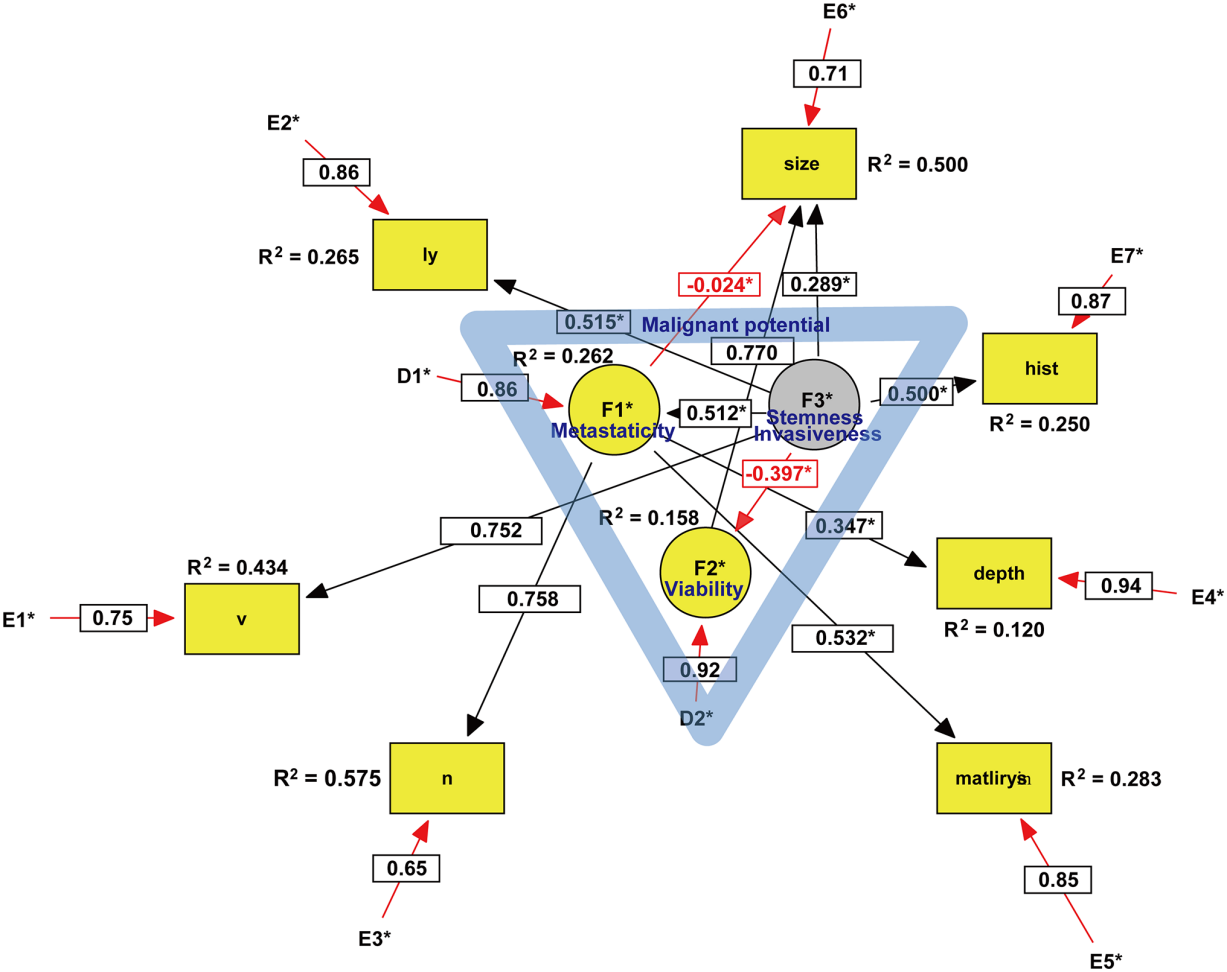
SEM of colorectal cancer. Circles indicate unobserved latent variables, while rectangles represent observed variables. Yellow indicates a dependent endogenous variable and grey indicates an independent exogenous variable. Significant paths with their estimated parameter are shown by solid lines, while insignificant paths are shown by broken lines (Fig 2). Red arrows represent either a negative causal effect or measurement errors within the model. The blue triangle in the center suggests that the malignant potential includes three latent variables. Coefficient of determination is written as R^2^. Abbreviations: v, vascular invasion; ly, lymphatic invasion; n, lymph node metastasis; hist, histological grade.

Non-recursive SEMs of GI cancers are depicted in Figs 1–3. The three latent variables, F1–3, were identified by exploratory factor analysis (EFA) with a direct oblimin rotation as a precursor to confirmatory factor analysis (CFA). The seven pathologic parameters, including ly, v, n, depth of penetration, matrilysin expression, tumor size, and histological grade, were introduced into the SEM analysis as observed variables. The model was statistically evaluated by *t*-tests of all paths, and *r*^2^ values or squared multiple correlations were calculated. Moreover, the model was modified by the multivariate Largrange Multiplier and Wald test [16]. There were no missing data in this SEM analysis. Detailed handling of binary data in ordinal or categorical variables, such as v, ly, n, depth, matrilysin expression, and grade, is not explained here but depends on modification of the ‘‘Lee- Poon-Bentler” approach using polychoric and polyserial correlation coefficients [17]. Statistical parameter estimation employed the maximally likelihood method with the ‘‘ROBUST” option implemented in the software [18] (See Text 1 in S1 File for details). To evaluate how well the model fit with the data, the comparative fit index (CFI), and root mean square error of approximation (RMSEA) were used as goodness-of-fit indices. According to conventional criteria, CFI >0.95 and RMSEA <0.08 indicate an acceptable fit, and CFI >0.97 and RMSEA <0.05 indicate a good fit [19]. Sample size was determined by a general rule of thumb that is that the minimum sample size should be preferably no less than 200 or 5–20 times the number of parameters to be estimated [20]. Power analysis of the SEM was performed using a web utility to generate the R code that can compute the statistical power for testing a covariance structure model using RMSEA [21]. All statistical analyses were conducted using EQS 6.2 (Multivariate Software, Inc., Encino, CA) [18], SPSS Statistics 21 (International Business Machines Corporation, Armonk, NY), and R (Available via http://www.R-project.org) [22]. Unfortunately, an extension of sensitivity analysis is not yet implemented in EQS.

## Results

### Characteristics of GI cancer patients

As shown in Table 1 in detail, ECA patients were predominantly male. The tumor size of CRC was significantly smaller than that of other GI cancers. ECA was likely to penetrate massively into the submucosal layer, whereas CRC was likely to penetrate slightly. GCA was likely to differentiate poorly compared with the other GI cancers. ECA invaded infrequently into the vasculature. Lymph node metastases were observed in ECA, GCA, and CRC, in that order of frequency.

Independent predictive factors for lymph node metastasis identified by logistic regression analysis were as follows: depth of penetration (*P* = 0.01), ly (*P* = 1.0E-4), and tumor matrilysin expression (*P* = 0.04) for ECA; depth of penetration (*P* = 0.02), ly (*P* = 4.5E-5), matrilysin (*P* = 0.01) for GCA; v (*P* = 0.03), and matrilysin (*P* = 1.44E-4) for CRC. It was noteworthy that matrilysin expression was the only negative predictor in GCA (Table 2).

**Table 2:** Predictive factors for lymph node metastasis in GI cancers.

Variable	Coefficient	Significance	Odds ratio	Odds ratio 95% CI
**ECA**				
** depth**	**2.189**	**0.01**	**8.92**	**2.55–31.200**
** ly**	**1.363**	**1.0E-4**	**3.91**	**1.96–7.77**
** matrilysin**	**0.995**	**0.04**	**2.71**	**1.38–5.31**
**GCA**				
** depth**	**2.629**	**0.02**	**13.86**	**1.50–128.28**
** ly**	**1.925**	**4.5E-5**	**6.85**	**2.72–17.29**
** matrilysin**	**-1.126**	**0.01**	**0.13**	**0.13–0.79**
**CRC**				
** v**	**1.579**	**0.03**	**4.85**	**1.69–13.92**
** matrilysin**	**2.295**	**1.44E-4**	**9.93**	**3.04–32.41**

Abbreviations: ECA, esophageal cancer; GCA, gastric cancer; CRC, colorectal cancer.

### Hypothesis I: Is malignant potential determined by common latent variables?

Because we did not have a substantial theoretical model concerning the undefined malignant potential of GI cancers, EFA was first performed for each type of GI cancer. As shown in Table 3, the scree test identified the three latent variables that we defined as key components of the malignant potential in each type of GI cancer. In ECA, two of the three latent variables designated as invasiveness and stemness were exogenous variables (Fig 1), whereas one of the three latent variables designated as stemness was an exogenous variable in GCA (Fig 2) and CRC (Fig 3). Because the exogenous (independent) variables were not affected by other variables, these results may reflect more autonomous progression of ECA than the other GI cancers. CFA was subsequently performed with direct oblimin rotation [23]. It was noteworthy that cancer stemness in both GCA and CRC negatively regulated cancer viability, whereas ECA stemness facilitated viability. Furthermore, GCA stemness could directly inhibit lymph node metastasis. These results suggested that GCA and CRC stemness partially maintain the normal stem cell niche property of tight control to prevent abnormal stem cells from proliferating uncontrollably. In contrast, ECA stemness lost such niche control and enhanced cancer viability. The malignant potential of ECA was more autonomous and higher than that of other GI cancers. In CRC, it was difficult to define the malignant potential by the same three variables in both ECA and GCA, and introduction of a new latent variable, ‘metastaticity’, was necessary. Consequently, there were substantial differences among the malignant potentials of the examined GI cancers.

**Table 3:** Malignant potential of each GI cancer type.

Latent Variable	ECA	GCA	CRC
**F1**	*invasiveness	**invasiveness**	**metastaticity**
**F2**	*stemness	*stemness	**viability**
**F3**	**viability**	**viability**	*stemness/invasiveness

Abbreviations: ECA, esophageal cancer; GCA, gastric cancer; CRC, colorectal cancer

Asterisks are exogenous variables. Others are endogenous variables.

### Hypothesis II: Is there substantial differences in the associations between malignant potential and pathologic parameters?

Because configural invariance across GI cancer groups was not approved, multigroup SEM [24] could not be performed, which strongly suggested that there were substantial differences in each type of GI cancer in the SEM. For example, in ECA, we could trace the three paths on the causal graph from a causal ancestor (invasiveness and stemness) to a causal descendant (lymph node metastasis): invasiveness → ly → n, invasiveness → depth → n, stemness → viability → matrilysin → n. In GCA, we could trace the only significant path from stemness to lymph node metastasis, which was likely to negatively regulate lymph node metastasis. In CRC, we could trace the only path from metastaticity to lymph node metastasis.

Based on power analysis of the SEM with an α-level of 0.05, the estimated power of ECA, GCA, and CRC SEM was calculated as 0.997, 0.997, and 0.678, respectively [21]. Goodness-of-fit indices of the established models were as follows: CFI = 1.000 and RMSEA = 0.000 in ECA, CFI = 0.974 and RMSEA = 0.065 in GCA, CFI = 0.909 and RMSEA = 0.113 in CRC.

## Discussion

We tested two hypotheses in this SEM. Hypothesis I is that the malignant potential of pT1 GI cancers is determined by three latent variables including stemness, invasiveness, and viability. Because the latent variables were disparate except for the number of variables in the examined GI cancers, hypothesis I was rejected. Despite this result, the SEM indicated that the malignant potential could be defined in combination with the latent variables introduced in the study. Hypothesis II is that there are substantial differences in the associations between malignant potential and pathological parameters according to the origin of the cancers. Because configural invariance across the GI cancer groups was not approved in a multigroup SEM analysis, this hypothesis was verified.

Unfortunately, despite many histological studies, we still cannot exactly assess the metastatic potential of cancer [25]. Assessment and prediction of the metastatic potential of cancer is difficult to evaluate directly. Fortunately, locoregional nodes are almost invariably the initial site of metastatic spread in pT1 GI cancers. Furthermore, pT1 GI cancers have a good prognosis compared to advanced stage disease. Therefore, the development of lymph node metastasis should reflect the metastatic and partly malignant potential of pT1 GI cancers. This is the rationale for the current study focusing on the malignant potential of pT1 GI cancers.

The goal of SEM analysis is to determine the extent to which the hypothesized model is supported by sample data. Although the current cross-sectional, observational data set cannot provide strong evidence of causation, one circumstance in which weak claims for causal inference can be made in the context of nonexperimental data is when there is temporal precedence between the predictor and criterion variables [26]. Because the predictor and criterion variables are correspond to the malignant potential represented by whole latent variables and lymph node metastasis (n), respectively, modest claims for causal inference between latent variables and lymph node metastasis can be made in our SEM analysis.

In ECA, we could trace the three paths on the causal graph from a causal ancestor (invasiveness and stemness) to a causal descendant (n), which were mediated through depth, ly, and matrilysin expression (Fig 1). Intriguingly, these parameters were in simultaneous accordance with predictive factors for lymph node metastasis identified by the logistic regression model (Table 2). In contrast to ECA, we could trace only a direct path from the latent variable to lymph node metastasis in both GCA and CRC, although the logistic regression model identified some predictive factors from observed variables. These results suggest that the causal inference of lymph node metastasis in both GCA and CRC largely remains to be clarified by further studies.

The malignant potential of ECA included two exogenous and one endogenous latent variables, whereas that of both GCA and CRC included one exogenous and two endogenous latent variables. Furthermore, cancer stemness promoted cancer cell viability in ECA, whereas it suppressed viability in both GCA and CRC. These results likely reflect a higher degree of malignant potential in ECA than the other GI cancers.

There are some limitations in this study. First, because evidence for causality is certainly the strongest in experimental designs with random assignment, further studies should be conducted with a longitudinal design. Second, in GCA, there were compellingly insignificant paths to lymph node metastasis as depicted by dotted lines in the developed model (Fig 2) because of ensuring goodness-of-fit in the established model [26]. Moreover, RMSEA in CRC was not less than 0.08. Further exploration of these models will be needed to resolve these issues. Third, it should be confirmed whether the authors’ latent variables were adequately designated by further SEM analyses. Fourth, clinicopathologic parameters, such as gender (S1 Table) and age (S2 Table), which were observed and exogenous variables, were excluded in this analysis because of avoiding complexity of the models and improper solutions. However, how gender or age affect the malignant potential of GI cancers in the other datasets warrants further SEM analyses, and this would be a very interesting question to be resolved in the near future. Fifth, statistical power more than 0.8 was required by recruiting more CRC patients. Finally, the clinical significance of tumor matrilysin expression remains to be clarified by further studies of GI cancers.

## Conclusion

In conclusion, we examined the challenging issue of assessing the malignant potential of GI cancers. We hope that such information contributes to refining clinical treatments for GI cancers.

## Supporting Information

S1 File Normal, Polychoric and Polyserial Correlations. (DOCX)

S1 Table Gender bias in the pathologic parameters. (DOCX)

S2 Table Age bias in the pathologic parameters. (DOCX)
