# Supplementary material for: Malignant Potential of Gastrointestinal Cancers Assessed by Structural Equation Modeling
Source: PLoS One. 2016 Feb 18;11(2):e0149327. doi: 10.1371/journal.pone.0149327 (PMC4758624; doi:10.1371/journal.pone.0149327)
Supplement: S1 File — (DOCX) [file pone.0149327.s001.docx]

**Text 1. Normal, Polychoric and Polyserial Correlations**

The standard statistical theory is that for means and covariances arising from continuous variables. If your data are normal, you can use normal theory correlation structure methods. These are based on Jennrich’s [1] statistical theory, also given by Shapiro and Browne [2], and extended in EQS to the usual least squares (LS), generalized least squares (GLS), and maximum likelihood (ML) methods. However, if your data are not normal, or you would like to estimate a structural model subsequent to estimating so-called polychoric and polyserial correlations, you should use the new extension of the Satorra-Bentler [3] robust methodology and robust standard errors [4], applied to correlation structures. You use some standard normal theory correlation estimator xx (xx= LS, GLS, or ML), and then the test statistic and standard errors of the estimator are corrected using the command METHOD=xx, ROBUST.

Polychoric correlations relate ordered categorical variables to each other, and polyserial correlations related ordered categorical and continuous variables. These correlations require special computations based on the assumption that a categorical variable is a categorized version of an underlying truly continuous, normally distributed, variable. EQS computes these correlations automatically by simply designating the categorical variables. EQS follows the tradition of Muthén [5], Jöreskog [6], and Lee, Poon and Bentler [7,8] of estimating a structural model subsequent to estimating these “poly” correlations [9].

# References

1. Jennrich RI. An Asymptotic χ 2 Test for the Equality of Two Correlation Matrices. J Am Stat Assoc. 1970;65: 904–912. doi:10.1080/01621459.1970.10481133

2. Shapiro A, Browne MW. On the treatment of correlation structures as covariance structures. Linear Algebra Appl. 1990;127: 567–587. doi:10.1016/0024-3795(90)90362-G

3. Satorra A, Bentler P. Corrections to test statistics and standard errors in covariance structure analysis. Latent variables analysis: applications for developmental research. London: Sage Publications, Inc; 1994. pp. 399–419.

4. Bentler, P. M. and Dijkstra T. “Efficient estimation via linearization in structural models” In: Krishnaiah PR, editor. Multivariate Analysis VI. Amsterdam: Elsevier Science Publisher; 1985. pp. 9–42.

5. Muthén B. A general structural equation model with dichotomous, ordered categorical, and continuous latent variable indicators. Psychom Soc. 1984;49: 115–132. doi:10.1007/BF02294210

6. Jöreskog KG. On the estimation of polychoric correlations and their asymptotic covariance matrix. Psychometrika. 1994;59: 381–389. doi:10.1007/BF02296131

7. Lee S, Poon W. A three-stage estimation procedure for structural equation models with polytomous variables. Psychometrika. 1990;55: 45–51.

8. Lee S-Y, Poon W-Y, Bentler PM. Structural equation models with continuous and polytomous variables. Psychometrika. 1992;57: 89–105. doi:10.1007/BF02294660

9. Bentler PM. EQS 6 structural equations program manual. Los Angeles: BMDP Statistic Software. 2006.
