# Supplementary material for: Malignant Potential of Gastrointestinal Cancers Assessed by Structural Equation Modeling
Source: PLoS One. 2016 Feb 18;11(2):e0149327. doi: 10.1371/journal.pone.0149327 (PMC4758624; doi:10.1371/journal.pone.0149327)
Supplement: S1 Table — (DOCX) [file pone.0149327.s002.docx]

**S1 Table. Gender bias in the pathologic parameters**

| Parameter | ECA (*P* value) | GCA (*P* value) | CRC (*P* value) |
| --- | --- | --- | --- |
| v | 0.610 | 0.610 | 0.177 |
| ly | 0.234 | 0.234 | 0.477 |
| n | 0.258 | 0.258 | 0.559 |
| depth | 0.644 | 0.699 | 0.267 |
| matrilysin | 0.388 | 0.388 | 0.448 |
| histology | 1.000 | 1.000 | 0.810 |
| size* | 0.711 | 0.401 | 0.256 |
| age* | 0.903 | 0.357 | 0.742 |

Cross table analysis with chi-square test except for analysis on gender bias in size and age. *Student *t* test
