# Supplementary material for: Malignant Potential of Gastrointestinal Cancers Assessed by Structural Equation Modeling
Source: PLoS One. 2016 Feb 18;11(2):e0149327. doi: 10.1371/journal.pone.0149327 (PMC4758624; doi:10.1371/journal.pone.0149327)
Supplement: S2 Table — (DOCX) [file pone.0149327.s003.docx]

**S2 Table. Age bias in the pathologic parameters**

| Parameter | ECA (*P* value) | GCA (*P* value) | CRC (*P* value) |
| --- | --- | --- | --- |
| v | 0.997 | 0.123 | 0.123 |
| ly | 0.659 | 0.472 | 0.472 |
| n | 0.434 | 0.783 | 0.783 |
| depth* |  |  |  |
| 1-2 | 0.996 | 0.961 | 0.670 |
| 1-3 | 0.530 | 0.922 | 0.969 |
| 2-3 | 0.321 | 0.981 | 0.846 |
| matrilysin | 0.143 | 0.172 | 0.985 |
| histology | 0.808 | 0.808 | 0.455 |
| size† | 0.207 | 0.316 | 0.923 |

Student *t* test was used except for analysis on age bias in depth and size. *One-way ANOVA followed by the Scheffe’s post-hoc test. †Significant test for Pearson product-moment correlation coefficient
